# Preoperative ctDNA and tumor volume predict colorectal cancer recurrence after metastasis resection

**DOI:** 10.1038/s41698-026-01450-w

**Published:** 2026-04-30

**Authors:** Hidekazu Oyoshi, Hideaki Bando, Riu Yamashita, Shun-Ichiro Kageyama, Yoshiaki Nakamura, Satoshi Horasawa, Masaki Nakamura, Takeshi Fujisawa, Kento Tomizawa, Atsushi Motegi, Hidehiro Hojo, Hidenari Hirata, Hiroki Yukami, Saori Mishima, Daisuke Kotani, Masaaki Miyo, Koji Ando, Jun Watanabe, Naoya Akazawa, Kozo Kataoka, Hiroya Taniguchi, Eiji Oki, Ichiro Takemasa, Takeshi Kato, Masaki Mori, Adham Jurdi, Minetta C. Liu, Toshihiro Misumi, Sadatomo Zenda, Takayuki Yoshino

**Affiliations:** 1 Department of Radiation Oncology, National Cancer Center Hospital East, Kashiwa, Japan; 2 Department of Gastroenterology and Gastrointestinal Oncology, National Cancer Center Hospital East, Kashiwa, Japan; 3 Department of Data Science, National Cancer Center Hospital East, Kashiwa, Japan; 4 Translational Research Support Office, National Cancer Center Hospital East, Kashiwa, Japan; 5 Division of Translational Informatics, Exploratory Oncology Research and Clinical Trial Center, National Cancer Center, Kashiwa, Japan; 6 Division of Radiation Oncology and Particle Therapy, Exploratory Oncology Research and Clinical Trial Center, National Cancer Center, Kashiwa, Japan; 7 The Second Department of Internal Medicine Center, Osaka Medical and Pharmaceutical University, Takatsuki, Japan; 8 Department of Gastroenterological Surgery, Osaka International Cancer Institute, Osaka, Japan; 9 Department of Surgery and Science, Graduate School of Medical Sciences, Kyushu University, Fukuoka, Japan; 10 Department of Colorectal Surgery, Kansai Medical University, Hirakata, Japan; 11 Department of Surgery, Gastroenterological Center, Yokohama City University Medical Center, Yokohama, Japan; 12 Department of Gastroenterological Surgery, Sendai City Medical Center, Sendai Open Hospital, Sendai, Japan; 13 Division of Lower Gastrointestinal, Department of Gastroenterological Surgery, Hyogo Medical University, Nishinomiya, Japan; 14 Department of Clinical Oncology, Aichi Cancer Center, Nagoya, Japan; 15 Department of Surgery, Osaka International Medical and Science Center, Osaka Keisatsu Hospital, Osaka, Japan; 16 Department of Surgery, National Hospital Organization Osaka National Hospital, Chuo-ku, Osaka, Japan; 17 Department of Gastroenterological Surgery, Tokai University School of Medicine, Isehara, Japan; 18 Natera, Inc., Austin, TX USA

**Keywords:** Cancer, Gastroenterology, Oncology

## Abstract

Circulating tumor DNA (ctDNA) testing in patients with colorectal cancer (CRC) has demonstrated clinical significance in various contexts, including post-curative resection. Therefore, we developed a predictive model integrating preoperative ctDNA levels with radiographic imaging to assess the risk of postoperative recurrence in patients with CRC. Patients with CRC from the GALAXY study with either newly diagnosed or recurrent, curatively resectable liver or lung metastases were enrolled between May 2020 and December 2022, and radiographic images were collected between February 2023 and January 2025. The ratio of preoperative ctDNA levels to tumor metastasis volume from radiographic imaging, ctDNA levels alone, and tumor volume alone was assessed. Overall, 181 and 48 patients from the GALAXY trial had liver and lung metastases, respectively. Among patients with liver metastases, the median progression-free survival (PFS) was 11.4 and 24 months in the high- and low-risk groups classified by the ctDNA model, respectively. Among patients with lung metastases, the median PFS was 12 months and not reached in the high- and low-risk groups classified by the ctDNA/volume model, respectively. Conclusively, incorporating radiological markers of necrosis and refining the tumor volume estimation may further improve model accuracy in liver metastasis settings.

## Introduction

Colorectal cancer (CRC) is the third most common cancer and the second-leading cause of cancer-related deaths globally^1^. Each year, more than 1.9 million people worldwide are newly diagnosed with CRC, and approximately 900,000 fatalities occur due to the disease. About 50–70% of patients with CRC develop metastases, significantly contributing to high mortality rates^2^. Local therapy, including surgical resection of resectable metastatic lesions, is the standard of care for patients with Stage IV or recurrent CRC^3,4^. However, approximately 40% of patients who undergo surgery for liver or lung metastases experience disease recurrence within 1 year^5,6^, highlighting the limited benefit and associated morbidity of surgical intervention in cases of early recurrence. Thus, there is an urgent need for pretreatment prognostic biomarkers to identify patients with resectable metastases who are at high risk of early recurrence, potentially sparing them from ineffective and invasive surgical interventions.

Circulating tumor DNA (ctDNA) consists of DNA fragments shed by tumors into the bloodstream. In patients with CRC, ctDNA testing has shown clinical relevance in various contexts, including post-curative resection^7^. While most studies have focused on the presence or absence of ctDNA to establish its clinical utility, limited research has investigated the clinical implications of actual ctDNA levels. The amount of ctDNA can be influenced by tumor size and the biological characteristics of metastases in specific organs^8^. For instance, ctDNA shedding appears to be higher in the liver than in the lung metastases in CRC^9^. In other tumor types, including early-stage non-small-cell lung cancer, combining tumor size and ctDNA status has been proposed to predict recurrence and survival^10^. Conversely, the preoperative utility of integrating ctDNA levels and tumor volume in resectable CRC remains unclear.

Hence, we hypothesized that elevated ctDNA levels relative to tumor volume might indicate a greater probability of subclinical micrometastases. Therefore, we aimed to develop a predictive model to estimate the risk of early postoperative recurrence before local therapy in patients with CRC with resectable liver or lung metastases. This approach seeks to reduce the morbidity associated with unnecessary procedures and expedite the initiation of effective systemic therapy.

## Results

### Patient selection and evaluation of chemotherapy effects on ctDNA levels

**Fig. 1:**
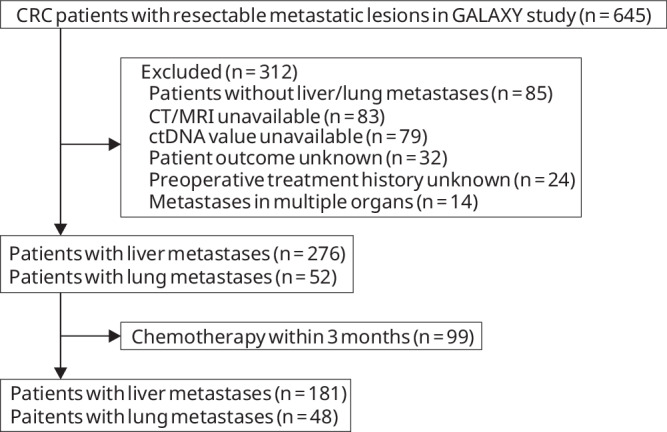
Consolidated Standards of Reporting Trials (CONSORT) diagram of patient inclusion in this study. CRC Colorectal cancer, CT Computed tomography, MRI Magnetic resonance imaging.

GALAXY Cohort C included 645 patients with CRC with resectable metastatic lesions (Fig. 1). Patients were excluded based on the following criteria: absence of liver or lung metastases (85 patients), missing CT or MRI data (83 patients), unavailable ctDNA levels (79 patients), unknown outcomes owing to immature data (32 patients), unknown preoperative treatment history (24 patients), and metastases involving multiple organs (14 patients). A total of 276 patients with liver metastases and 52 patients with lung metastases were included in the analysis following these exclusions.

We evaluated PFS after surgery by comparing the preoperative ctDNA level (MTM). Prior to the exclusion of patients who underwent chemotherapy within 3 months preceding ctDNA measurement, the median PFS of patients with liver and lung metastases was 14.1 months (95% CI: 11.0–17.6) and 19.6 months (95% CI: 15.3–not reached), respectively.

To minimize confounding effects on interpreting ctDNA levels, we investigated the impact of the interval between the last day of chemotherapy administration and the date of ctDNA measurement (Table 1). This analysis revealed that excluding patients who received chemotherapy within 3 months before ctDNA assessment led to a more accurate prognostic prediction in the liver metastasis groups (*P* = 0.011). In the lung metastasis group, excluding patients who received chemotherapy within 1 month before ctDNA assessment resulted in the weakest association (*P* = 0.022). The strongest association was observed when patients who received chemotherapy within 4 months were excluded (*P* = 0.0058); however, the number of patients differed by only one compared with the 2–3-month (*P* = 0.013) and 5-month (P = 0.0088) groups. Consequently, 99 patients who received chemotherapy within 3 months were excluded owing to the potential impact of recent chemotherapy on ctDNA levels. No patients received immune checkpoint inhibitors prior to metastatic resection. The median follow-up of the remaining 181 patients with liver metastases and 48 patients with lung metastases was 22.3 months (range: 0.2–47.9 months) from the date of surgery.

**Table 1:** Evaluation of the ctDNA model and effects of chemotherapy and cutoff value for each patient group

Patient group predicted by ctDNA model	Liver (*n* = 276)		Lung (*n* = 52)	
	*P*-value^a^	Cutoff value^b^	*P*-value^a^	Cutoff value^b^
All patients	0.68 (*n* = 276)	0.725	0.018 (*n* = 52)	0.045
Patients without chemotherapy within 1 month prior to ctDNA measurement	0.018 (*n* = 236)	85.48	0.022 (*n* = 51)	0.045
Patients without chemotherapy within 2 months prior to ctDNA measurement	0.016 (*n* = 194)	85.48	0.013 (*n* = 48)	0.045
Patients without chemotherapy within 3 months prior to ctDNA measurement	0.011 (*n* = 181)	85.48	0.013 (*n* = 48)	0.045
Patients without chemotherapy within 4 months prior to ctDNA measurement	0.013 (*n* = 176)	85.48	0.0058 (*n* = 47)	0.045
Patients without chemotherapy within 5 months prior to ctDNA measurement	0.043 (*n* = 168)	85.48	0.0088 (*n* = 46)	0.045
Patients without chemotherapy within 6 months prior to ctDNA measurement	0.052 (*n* = 160)	85.48	0.037 (*n* = 43)	0.045

^a^ *P*-values were calculated using Generalized Wilcoxon test.

^b^ Cutoff values were calculated using receiver operating characteristic curves and the Youden index.

### Comparisons of the ctDNA/volume, ctDNA, and volume models

In patients with lung metastases, the ctDNA/volume model was a superior predictor of PFS (*P* = 0.000017, hazard ratio [HR] = 5.5), outperforming the ctDNA (*P* = 0.013, HR = 2.7) and tumor volume models (*P* = 0.12, owing to the small number of high-risk patients [*n* = 3] with no events, the HR estimate was unstable) (Table 2). In contrast, among patients with liver metastases, the ctDNA model was a superior predictor of PFS (*P* = 0.011, HR = 1.6), outperforming the ctDNA/volume (*P* = 0.53, HR = 0.8) and volume models (*P* = 0.052, HR = 1.6).

**Table 2:** Comparison of ctDNA/volume, ctDNA, and volume models

Patient group predicted by each model	ctDNA/volume	ctDNA	Volume
Liver (*n* = 181)	*P* = 0.53, HR = 0.8	*P* = 0.011, HR = 1.6	*P* = 0.052, HR = 1.6
Lung (*n* = 48)	*P* < 0.0001, HR = 5.5	*P* = 0.013, HR = 2.7	*P* = 0.12, HR = 0

*P*-values were calculated using Generalized Wilcoxon test.

### Association of the ctDNA and ctDNA/volume models with PFS after surgery

We identified cutoff values of 85.48 MTM/mL for the ctDNA model in patients with liver metastases and 0.474 MTM/mL^2^ for the ctDNA/volume model in patients with lung metastases (areas under the curve were 0.56 and 0.69, respectively) (Supplementary Fig. S1). Based on these thresholds, 120 high-risk and 61 low-risk patients were identified among those with liver metastases, including 34 high-risk and 14 low-risk patients among those with lung metastases. Patient characteristics stratified by risk status are detailed in Supplementary Table S1 (liver metastases) and Supplementary Table S2 (lung metastases). Notably, among patients with liver metastases, the metastatic tumor volume was significantly lower in the low-risk group than in the high-risk group (*P* = 0.00000083).

**Fig. 2:**
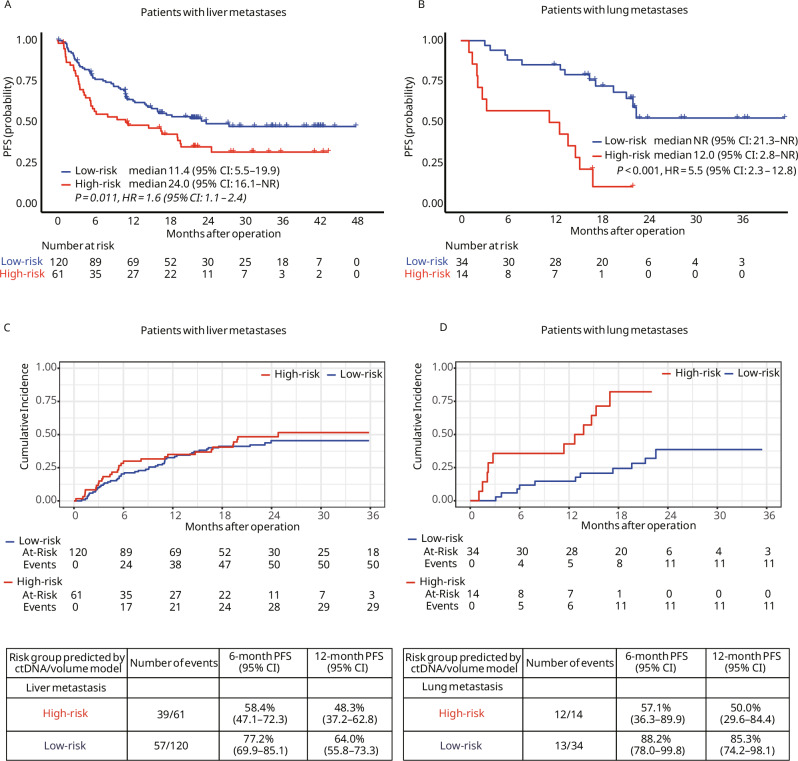
Progression-free survival and cumulative incidence according to risk group in colorectal cancer patients with liver and lung metastases after resection. **A, B** Progression-free survival (PFS) in patients with colorectal cancer (CRC) with liver **A** and lung **B** metastases after resection according to classified risk group. **C**, **D** Cumulative incidence in patients with CRC with liver **C** and lung **D** metastases after resection according to classified risk group. CI: Confidence of interval, HR: Hazard ratio, NR: Not reached.

Among patients with liver metastases, those classified as high-risk by the ctDNA model exhibited a median PFS of 11.4 months (95% confidence interval [CI]: 5.5–19.9), compared with 24 months (95% CI: 16.1–not reached) in the low-risk group (HR = 2.4, *P* = 0.011) (Fig. 2a). Similarly, among patients with lung metastases, the high-risk group had a median PFS of 12 months (95% CI: 2.8–not reached), significantly shorter than that observed in the low-risk group (95% CI: 21.3–not reached) (HR = 5.5, *P* = 0.000017) (Fig. 2b). The OS of patients with liver metastases classified as high-risk by the ctDNA model was significantly worse (not reached, 95% CI: not reached–not reached) compared with that of those classified as low-risk (not reached, 95% CI: not reached–not reached) (HR = 3.2, *P* = 0.010) (Supplementary Fig. S2a). In contrast, among patients with lung metastases, no clear difference was observed in OS between the high- and low-risk groups classified by the ctDNA/volume model, likely owing to the short follow-up period and the small number of events (Supplementary Fig. S2b).

Considering death as a competing risk, patients in the high-risk groups for liver and lung metastases exhibited higher cumulative incidences of recurrence at 12 months (36% and 43% in patients with liver and lung metastases, respectively) than those in the low-risk group (31%, *P* = 0.48; and 15%, *P* = 0.00031 in patients with liver and lung metastases, respectively) (Fig. 2c, d).

### Relationship between postoperative ctDNA status and postoperative PFS

Finally, we further stratified the patients with CRC according to their ctDNA status at 4 weeks postoperatively, in addition to the preoperative high- and low-risk classification using the ctDNA and ctDNA/volume models. Six patients with liver metastases lacking postoperative ctDNA data were excluded. Among the remaining 175 patients, 54.2% (32/59) classified as high-risk group according to the ctDNA model were ctDNA-positive, whereas 45.8% (27/59) were ctDNA-negative. In the low-risk group, 27.6% (32/116) were ctDNA-positive and 72.4% (84/116) were ctDNA-negative.

**Fig. 3:**
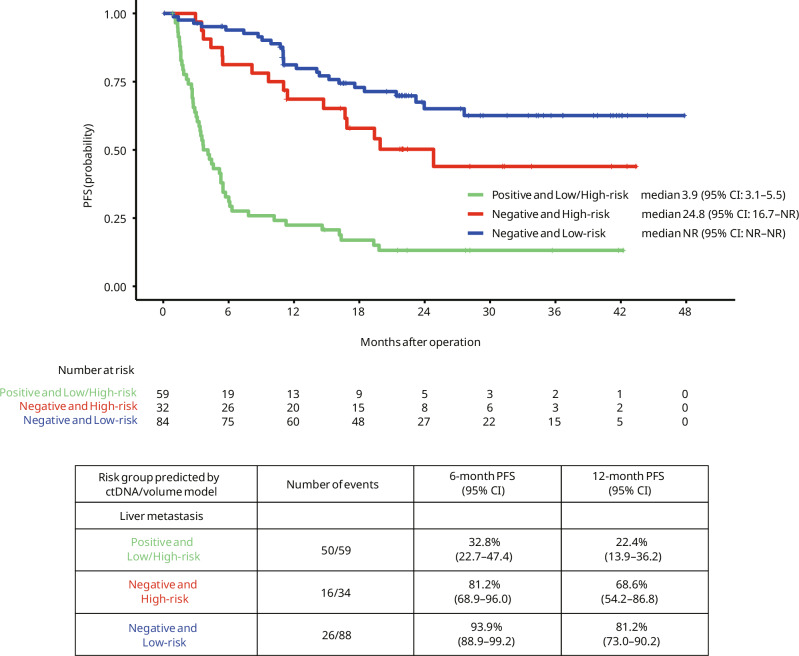
Progression-free survival according to circulating tumor DNA status in colorectal cancer patients with liver metastases after resection. Progression-free survival (PFS) of patients with colorectal cancer (CRC) with liver metastases after resection according to circulating tumor DNA (ctDNA) status 4 weeks postoperatively according to classified risk group. CI: Confidence of interval, NR: Not reached.

Most patients with liver metastases with postoperative ctDNA positivity experienced recurrence or death from CRC (84.7%), with a median PFS of 3.9 months (95% CI: 3.1–5.5) (Fig. 3). In the postoperative ctDNA-negative groups, the median PFS was 24.8 months for the high-risk group, while the low-risk group did not reach a median PFS (95% CI: 16.7 months–not reached for the high-risk group and not reached–not reached for the low-risk group). The proportion of patients experiencing recurrences was higher in the high-risk ctDNA-negative group than in the low-risk ctDNA-negative group (50.0% vs. 33.0%, respectively) (Fig. 3).

Among patients with lung metastases, two without postoperative ctDNA data were excluded. Among the remaining 46 patients, 11 high-risk patients (78.6%) and one low-risk patient (2.9%) had postoperative ctDNA positivity, while three high-risk (21.4%) and 33 low-risk patients (97.1%) had postoperative ctDNA negativity. The median PFS of patients with postoperative ctDNA positivity was 3 months (95% CI: 2.1–not reached) (Supplementary Fig. S3). In the postoperative ctDNA-negative groups, the median PFS was 17 months (95% CI: 15.3 months–not reached) for the high-risk group, while it was not reached (95% CI: 21.3 months–not reached) for the low-risk group (Supplementary Fig. S3).

## Discussion

This study’s results demonstrate that the preoperative ctDNA and ctDNA/volume models effectively predict postoperative recurrence after surgery for liver and lung metastatic lesions, respectively, in patients with CRC. These findings indicate that the ctDNA/tumor volume model can be utilized to prevent potentially ineffective surgery in patients at risk of early postoperative recurrence. To our knowledge, this is the first study to demonstrate the clinical utility of evaluating both the quantitative levels of ctDNA and tumor volume in resectable metastases from solid tumors.

Studies evaluating the prognostic significance of preoperative ctDNA in patients undergoing resection of metastatic lesions from CRC remain limited. Specifically, for liver metastases from CRC, multiple studies suggest that preoperative ctDNA status alone has limited ability to predict recurrence-free survival or OS^11–13^. While, Kobayashi et al. reported that preoperative ctDNA positivity was associated with shorter RFS in patients with solitary colorectal liver metastases; however, their cohort included patients who received preoperative chemotherapy^14^. Conversely, Liu et al. demonstrated that ctDNA negativity after preoperative chemotherapy was associated with prolonged RFS in patients with colorectal liver metastasis^15^. In contrast to these studies, the current study was designed to minimize the influence of preoperative chemotherapy when evaluating the prognostic significance of preoperative ctDNA. Furthermore, to the best of our knowledge, no previous studies have evaluated the prognostic significance of preoperative ctDNA in patients undergoing resection of lung metastases from CRC.

We considered the effects of preoperative chemotherapy and the metastatic sites to evaluate the clinical utility of our models accurately. Preoperative chemotherapy is commonly administered to patients with CRC exhibiting resectable metastatic lesions, resulting in decreased ctDNA levels; this reduction complicates the accurate evaluation of the relationship between ctDNA levels and clinical outcomes^16^. Furthermore, the duration of ctDNA reduction following chemotherapy remains unclear. In this study, we categorized patients based on the interval between the last chemotherapy dose and the blood drawn for ctDNA measurement, assessing intervals up to 6 months to assess the potential influence of chemotherapy on ctDNA levels. Compared with the liver metastasis group, the number of patients in the lung metastasis group was smaller, and the difference in the number of excluded patients between the 2-month and 5-month intervals was only two. However, these findings suggest that chemotherapy administered within 2 months before ctDNA assessment may influence ctDNA levels. Considering this and the results observed in the the liver metastasis group, we determined that excluding patients who had received chemotherapy within 3 months before ctDNA assessment would be appropriate. Another consideration is the variation in ctDNA levels according to the metastatic site^9^. To address this, we grouped patients with CRC by metastatic site and assessed their ctDNA levels separately. These stratifications allowed exclusion of confounding factors affecting ctDNA levels, thereby ensuring a more accurate assessment of the relationship between ctDNA levels and tumor volume. To apply our model in clinical practice, routine ctDNA measurement before initiating preoperative chemotherapy is necessary. Postoperative ctDNA has been reported as a predictive factor for recurrence after CRC resection surgery and plays a crucial role in postoperative follow-up. However, identifying the patient group most likely to benefit from resection surgery preoperatively also holds significant clinical value. This could be particularly important in healthcare systems where careful patient selection is essential.

This study has some limitations. First, the accuracy of the radiological evaluations may not be entirely reliable because of human error. In this study, one patient with liver metastases, categorized as low-risk, had postoperative ctDNA positivity and experienced recurrence within 1.6 months, ultimately dying at 10.7 months. This patient was initially reported to have two liver metastases; however, preoperative contrast-enhanced CT identified three additional lesions with contrast-enhancement patterns consistent with known metastatic lesions, suggesting the possibility of undetected metastases. This underscores the importance of a thorough radiographic evaluation to identify all metastatic lesions when applying this model. Second, necrosis within tumors may affect the assessment of ctDNA levels. In this study, two patients classified in the high-risk group for liver metastases exhibited notably high ctDNA levels (>10,000 MTM/mL). Radiographic findings suggested necrosis in these cases, aligning with prior findings showing that ctDNA concentrations tend to increase in tumors associated with necrosis^17^. Despite their high-risk classification, these two patients did not experience recurrence, suggesting that necrotic factors detected on contrast-enhanced CT scans should be considered in future models. Third, the cutoff values used to define risk groups were determined using ROC analysis within the same cohort used for outcome analysis. Therefore, the predictive performance of the model may be overestimated, and external validation in an independent cohort is warranted. Finally, variability may arise from individual contouring and the choice of radiographic modality. In this study, a radiation oncologist experienced in tumor contouring for stereotactic body radiotherapy contoured the metastatic lesions to minimize discrepancies in tumor volume assessment. Volume measurements of liver metastases may differ between CT and MRI images, and the use of contrast agents may further affect lesion contour delineation. Moreover, the lesions were primarily contoured using plain CT images to prevent the variability introduced by the timing of contrast injection and image acquisition, which can influence the apparent condition of the tumor. However, these issues can be addressed through the implementation of artificial intelligence (AI)-based automated detection of metastatic lesions. Incorporating AI-based approaches into predictive models could enhance the integration of qualitative factors (such as tumor necrosis) with ctDNA levels and tumor volume, thereby improving the accuracy of automated tumor contouring.

In this study, the ctDNA/volume model successfully predicted prognosis in patients with CRC with lung metastases, but not in those with liver metastases. One factor affecting the volume of liver metastasis tumors is that, while the boundaries of lung metastasis lesions are clear, those of liver metastasis lesions can be unclear. Liver metastases also have the potential to cause necrosis, a feature not observed in lung metastases, which may have led to excessive ctDNA detection in the liver metastasis group. Moreover, ctDNA shedding may have been higher in liver metastases than in lung metastases. Differences in the tumor microenvironment between liver and lung metastases of CRC may also influence the relationship between tumor volume and ctDNA levels^18^. If these issues can be addressed through technological advancements, the ctDNA/volume model may become more useful than the ctDNA model, as demonstrated in patients with lung metastases.

In conclusion, preoperative ctDNA levels and tumor metastasis volume successfully predicted postoperative recurrence in patients with CRC with liver and lung metastases. This model is beneficial for identifying patients with CRC with resectable metastatic lesions who are likely to experience early postoperative recurrence, thereby preventing potentially unnecessary surgical interventions. Ongoing and future studies with increased sample size will further optimize the model by incorporating other clinicopathologic factors associated with postoperative CRC recurrence.

## Methods

### Study design and patients

This research was conducted as an additional analysis within the framework of the GALAXY study, a prospective, large-scale, nationwide registry designed to monitor ctDNA status in patients diagnosed with clinical stage I–IV CRC who are eligible for complete surgical resection^19^.

We selected patients with CRC from the GALAXY study with either newly diagnosed or recurrent, curatively resectable liver or lung metastases (Cohort C). Patients were enrolled between May 2020 and December 2022. Radiographic images were retrospectively collected from participating institutions between February 2023 and January 2025 following an amendment to the Institutional Review Board approval in December 2022. Patients were excluded if they met the following criteria: unknown preoperative treatment history, unavailable ctDNA levels, absence of liver or lung metastases, unknown patient outcome, lack of computed tomography (CT) or magnetic resonance imaging (MRI) data, or metastases in multiple organs.

### Ethical approval

Written informed consent was obtained from all participants before enrollment. The clinical protocol was approved by the Institutional Review Board of the National Cancer Center, Japan, and authorized by the director of each participating institution (Protocol Number 2019-206). The study was registered in the Japan Registry of Clinical Trials (UMIN000039205) and was conducted in accordance with the principles of the Declaration of Helsinki.

### Assessment of ctDNA level and metastatic tumor volume

A clinically validated, personalized, tumor-informed 16-plex polymerase chain reaction -next-generation sequencing assay (SignateraTM, Natera, Inc.) was used to detect and quantify ctDNA in blood samples, as previously described^7^. Briefly, whole-exome sequencing of resected tumor tissue and matched normal DNA from each patient was utilized to design patient-specific ctDNA assays for tracking up to 16 single-nucleotide variants. Plasma samples containing at least 2 of the 16 variants with a confidence score above a predefined algorithm threshold were denoted as ctDNA-positive. Plasma samples deemed ctDNA-positive were quantified, and the ctDNA concentrations were reported as mean tumor molecules (MTM) per milliliter of plasma. Preoperative ctDNA was measured within 4 weeks preoperatively, while postoperative ctDNA was assessed 4 weeks after surgery.

Metastatic lesions were defined based on radiologists’ interpretations. Tumor metastasis volume was evaluated by a radiation oncologist, who contoured the metastatic lesions on CT or MRI (with or without contrast enhancement) using the Eclipse Treatment Planning System version 16.1 (Varian Medical Systems, Palo Alto, CA, USA). CT or MRI was performed immediately preoperatively to confirm resectability, and volume of metastatic lesions was measured using these preoperative images. All tumor contours were reviewed and verified by a second radiation oncologist. Tumor metastasis volume was defined as the sum of the volumes of all metastatic lesions in the liver and lungs, respectively.

### Data analysis

Considering the greater impact of liver metastases on ctDNA levels than that of lung metastases, we analyzed patients with liver and lung metastases separately. Furthermore, as the use of chemotherapy before ctDNA measurement can alter ctDNA levels^20^, we categorized patients based on the interval between the last day of chemotherapy administration and the date of preoperative ctDNA level assessment. The analysis was performed on the entire cohort and was also performed after sequentially excluding patients who received chemotherapy 1 month, 2 months, and 3 months prior to ctDNA measurement. This stratification allowed us to accurately evaluate progression-free survival (PFS) for each group, minimizing confounding effects from recent chemotherapy.

To predict recurrence, we developed and assessed three models: ctDNA levels alone (ctDNA model), tumor metastasis volume alone (volume model), and the ratio of preoperative ctDNA levels to tumor metastasis volume (ctDNA/volume model). Cutoff values to classify patients into high- and low-risk groups for recurrence were determined using receiver operating characteristic (ROC) curves and the Youden index. Further, PFS of the high- and low-risk groups were compared using these cutoff values and evaluated which model had the strongest prognostic performance.

We employed the Kaplan–Meier method to estimate PFS and overall survival (OS) based on short-term follow-up and compared survival curves between the high- and low-risk groups using Gehan’s generalized Wilcoxon method. OS was calculated from the date of surgery for the metastatic lesions to the date of death from any cause or the latest clinical follow-up. PFS was calculated from the date of surgery for the metastatic lesions to the date of death or disease progression, whichever occurred first, or the latest clinical follow-up. Follow-up was censored in April 2023.

### Statistical analysis

Statistical significance was set at *P* < 0.05. All analyses were conducted using R software version 4.4.2.

### Consent for publication

Written informed consent for study participation and secondary use of data and samples was obtained from all patients enrolled in the GALAXY study.

## Supplementary information

Supplementary information

## Data Availability

The datasets generated and/or analyzed during the current study are not publicly available due to institutional regulations but are available from the corresponding author on reasonable request.

## References

[1] Bray, F. et al. Global cancer statistics 2022: GLOBOCAN estimates of incidence and mortality worldwide for 36 cancers in 185 countries. *CA Cancer J. Clin.* **74**, 229–263 (2024). 38572751 10.3322/caac.21834

[2] Adam, R. & Kitano, Y. Multidisciplinary approach of liver metastases from colorectal cancer. *Ann. Gastroenterol. Surg.* **3**, 50–56 (2019). 30697610 10.1002/ags3.12227 PMC6345652

[3] Network NCC. Colon Cancer (Version 1.2024). URL: https://www.nccn.org/professionals/physician_gls/pdf/colon.pdf. (Accessed 1 March 2024).

[4] Network NCC. Rectal Cancer (Version 1.2024). URL: https://www.nccn.org/professionals/physician_gls/pdf/rectal.pdf. (Accessed 1 March 2024).

[5] Kanemitsu, Y. et al. Hepatectomy followed by mFOLFOX6 versus hepatectomy alone for liver-only metastatic colorectal cancer (JCOG0603): a phase II or III randomized controlled trial. *J. Clin. Oncol.* **39**, 3789–3799 (2021). 34520230 10.1200/JCO.21.01032

[6] Okumura, T. et al. Surgical outcome and prognostic stratification for pulmonary metastasis from colorectal cancer. *Ann. Thorac. Surg.* **104**, 979–987 (2017). 28577846 10.1016/j.athoracsur.2017.03.021

[7] Kotani, D. et al. Molecular residual disease and efficacy of adjuvant chemotherapy in patients with colorectal cancer. *Nat. Med.* **29**, 127–134 (2023). 36646802 10.1038/s41591-022-02115-4 PMC9873552

[8] Strijker, M. et al. Circulating tumor DNA quantity is related to tumor volume and both predict survival in metastatic pancreatic ductal adenocarcinoma. *Int. J. Cancer* **146**, 1445–1456 (2020). 31340061 10.1002/ijc.32586 PMC7004068

[9] Bando, H. et al. Effects of metastatic sites on circulating tumor DNA in patients with metastatic colorectal cancer. *JCO Precis. Oncol.* **6**, e2100535 (2022). 35544728 10.1200/PO.21.00535

[10] Tran, H. T. et al. Circulating tumor DNA and radiological tumor volume identify patients at risk for relapse with resected, early-stage non-small-cell lung cancer. *Ann. Oncol.* **35**, 183–189 (2024). 37992871 10.1016/j.annonc.2023.11.008 PMC11233158

[11] Newhook, T. E. et al. Prospective study of perioperative circulating tumor DNA dynamics in patients undergoing hepatectomy for colorectal liver metastases. *Ann. Surg.* **277**, 813–820 (2023). 35797554 10.1097/SLA.0000000000005461 PMC9816346

[12] Øgaard, N. et al. Tumour-agnostic circulating tumour DNA analysis for improved recurrence surveillance after resection of colorectal liver metastases: a prospective cohort study. *Eur. J. Cancer* **163**, 163–176 (2022). 35074652 10.1016/j.ejca.2021.12.026

[13] Wang, D.-S. et al. Dynamic monitoring of circulating tumor DNA to predict prognosis and efficacy of adjuvant chemotherapy after resection of colorectal liver metastases. *Theranostics* **11**, 7018 (2021). 34093868 10.7150/thno.59644 PMC8171084

[14] Kobayashi, S. et al. Impact of preoperative circulating tumor DNA status on survival outcomes after hepatectomy for resectable colorectal liver metastases. *Ann. Surg. Oncol.* **28**, 4744–4755 (2021). 33393041 10.1245/s10434-020-09449-8

[15] Liu, M. et al. Pre-hepatectomy dynamic circulating tumor DNA to predict pathologic response to preoperative chemotherapy and post-hepatectomy recurrence in patients with colorectal liver metastases. *Hepatol. Int.* **18**, 1029–1039 (2024). 38427145 10.1007/s12072-023-10628-4

[16] Schraa, S. J., van Rooijen, K. L., Koopman, M., Vink, G. R. & Fijneman, R. J. A. Cell-free circulating (tumor) DNA before surgery as a prognostic factor in non-metastatic colorectal cancer: a systematic review. *Cancers (Basel)* **14**, 2218 (2022). 35565347 10.3390/cancers14092218 PMC9101623

[17] Siravegna, G., Marsoni, S., Siena, S. & Bardelli, A. Integrating liquid biopsies into the management of cancer. *Nat. Rev. Clin. Oncol.* **14**, 531–548 (2017). 28252003 10.1038/nrclinonc.2017.14

[18] Chandra, R. et al. The colorectal cancer tumor microenvironment and its impact on liver and lung metastasis. *Cancers* **13**, 6206 (2021). 34944826 10.3390/cancers13246206 PMC8699466

[19] Taniguchi, H. et al. CIRCULATE-Japan: circulating tumor DNA–guided adaptive platform trials to refine adjuvant therapy for colorectal cancer. *Cancer Sci.* **112**, 2915–2920 (2021). 33931919 10.1111/cas.14926 PMC8253296

[20] Osumi, H., Shinozaki, E., Yamaguchi, K. & Zembutsu, H. Early change in circulating tumor DNA as a potential predictor of response to chemotherapy in patients with metastatic colorectal cancer. *Sci. Rep.* **9**, 17358 (2019). 31758080 10.1038/s41598-019-53711-3 PMC6874682

