# Supplementary material for: Preoperative ctDNA and tumor volume predict colorectal cancer recurrence after metastasis resection
Source: NPJ Precis Oncol. 2026 Apr 30;10:259. doi: 10.1038/s41698-026-01450-w (PMC13338026; doi:10.1038/s41698-026-01450-w)
Supplement: Supplementary file 1 — Supplementary information [file 41698_2026_1450_MOESM1_ESM.pdf]

Supplementary Materials for

Preoperative ctDNA and tumor volume predict colorectal cancer recurrence after metastasis resection

Hidekazu Oyoshi, Hideaki Bando, Riu Yamashita, Shun-Ichiro Kageyama, Yoshiaki Nakamura, Satoshi Horasawa, Masaki Nakamura, Takeshi Fujisawa, Kento Tomizawa, Atsushi Motegi, Hidehiro Hojo, Hidenari Hirata, Hiroki Yukami, Saori Mishima, Daisuke Kotani, Masaaki Miyo, Koji Ando, Jun Watanabe, Naoya Akazawa, Kozo Kataoka, Hiroya Taniguchi, Eiji Oki, Ichiro Takemasa, Takeshi Kato, Masaki Mori, Adham Jurdi, Minetta C. Liu, Toshihiro Misumi, Sadatomo Zenda, Takayuki Yoshino

***Corresponding authors:**

Correspondence: hbando@east.ncc.go.jp (H.B.)

Correspondence: skageyam@east.ncc.go.jp (S.K.)

The PDF file includes:

Figure S1 to S3

Table S1 and S2

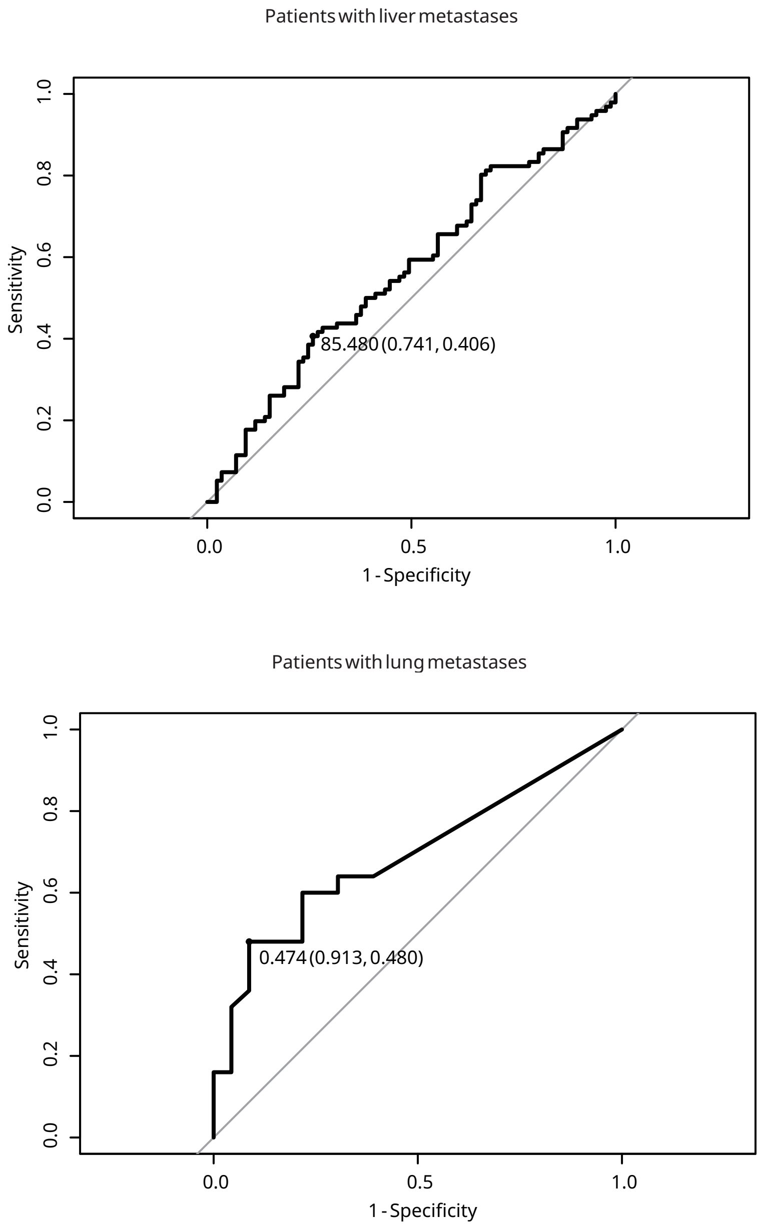

Figure S1. Receiver operating characteristic curves of the circulating tumor DNA (ctDNA)/volume model for patients with colorectal cancer with liver and lung metastases.

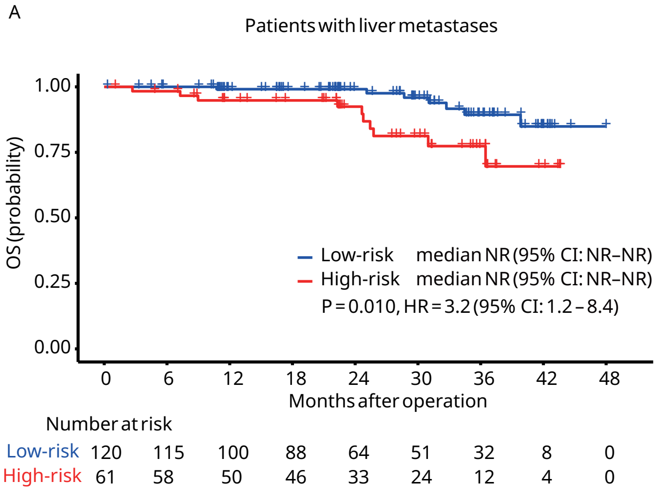

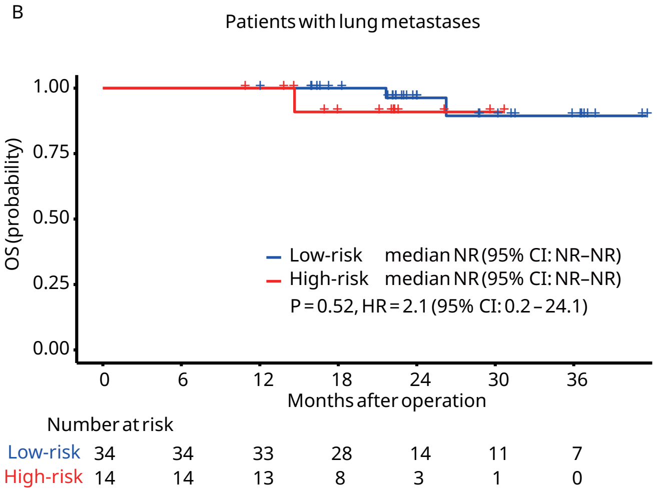

Risk group predicted by ctDNA/volume model	Number of events	6-month OS (95% CI)	12-month OS (95% CI)
Liver metastasis			
High-risk	10/61	100% (100–100)	99.1% (97.4–100)
Low-risk	7/120	98.3% (95.2–100)	94.8% (89.3–100)

Risk group predicted by ctDNA/volume model	Number of events	6-month OS (95% CI)	12-month OS (95% CI)
Lung metastasis			
High-risk	1/14	100% (100–100)	100% (100–100)
Low-risk	2/34	100% (100–100)	100% (100–100)

Figure S2. A, B, Overall survival (OS) in patients with colorectal cancer with liver (A) and lung (B) metastases after resection according to classified risk group. NR: not reached; CI: confidence interval.

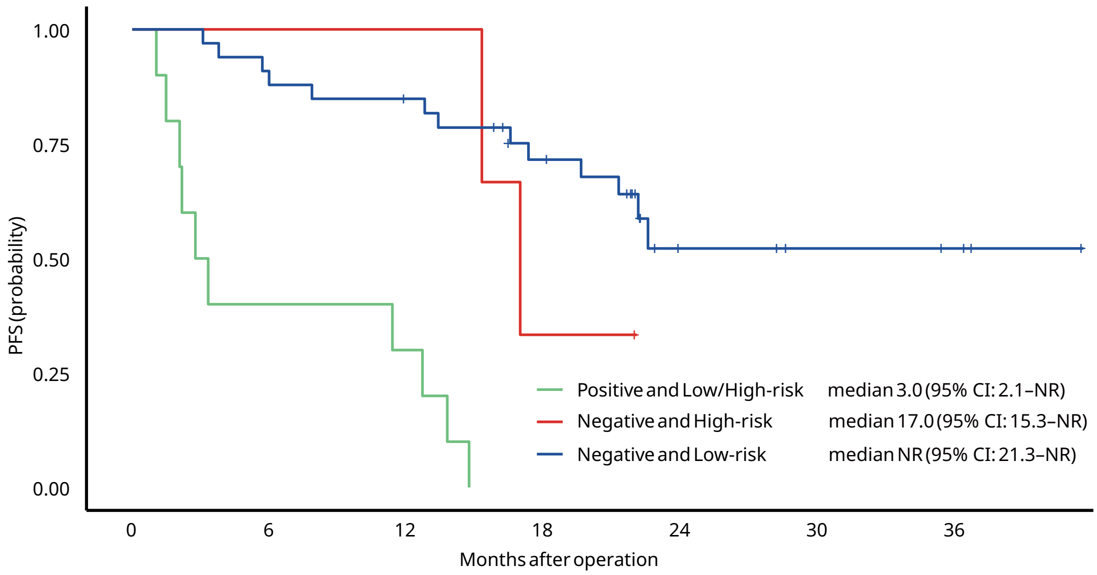

Number at risk

Positive and Low/High-risk	10	4	3	0	0	0	0
Negative and High-risk	3	3	3	1	0	0	0
Negative and Low-risk	33	29	27	20	6	4	3

Risk group predicted by ctDNA/volume model	Number of events	6-month PFS (95% CI)	12-month PFS (95% CI)
Lung metastasis			
Positive and Low/High-risk	10/10	40.0% (18.7–85.5)	30.0% (11.6–77.3)
Negative and High-risk	2/3	100% (0–100)	100% (0–100)
Negative and Low-risk	13/33	94.1% (77.4–99.8)	87.9% (73.5–98.0)

Figure S3. Progression-free survival (PFS) of patients with colorectal cancer with lung metastases after resection according to circulating tumor DNA (ctDNA) status four weeks postoperatively according to classified risk group. NR: not reached, NA: not available.

Table S1. Characteristics of patients with liver metastases

	Risk		
**Characteristics**	**Low-risk (n = 120)** [1]	**High-risk (n = 61)**[1]	***P*-value**[2]
**Age (year)**	65 (57, 73)	69 (59, 73)	0.36
**Sex**			0.87
F	39 (32.5%)	21 (34.4%)	
M	81 (67.5%)	40 (65.6%)	
**ECOG PS**			> 0.99
0	117 (97.5%)	59 (96.7%)	
1	3 (2.5%)	2 (3.3%)	
**Primary lesion**			0.61
Absent	90 (86.5%)	50 (90.9%)	
Present	14 (13.5%)	5 (9.1%)	
Unknown	16	6	
**RAS mutation**			0.63
Absent	66 (55.5%)	36 (60.0%)	
Present	53 (44.5%)	24 (40.0%)	
Unknown	1	1	
**BRAF V600E mutation**			0.60
Absent	117 (98.3%)	58 (96.7%)	
Present	2 (1.7%)	2 (3.3%)	
Unknown	1	1	
**MSI-High**			> 0.99
Absent	115 (99.1%)	57 (98.3%)	
Present	1 (0.9%)	1 (1.7%)	
Unknown	4	3	
**Liver metastasis volume**	3.5 (1.7, 6.6)	16.4 (5.6, 33.1)	< 0.001

ECOG PS: Eastern Cooperative Oncology Group performance status

[1]Median (interquartile range)

[2]Wilcoxon rank sum test or Fisher's exact test

Table S2. Characteristics of patients with lung metastases

	Risk		
**Characteristics**	**Low-risk (n = 34)**[1]	**High-risk (n = 14)**[1]	***P*-value**[2]
**Age(year)**	67 (58, 72)	68 (66, 72)	0.71
**Sex**			0.024
F	21 (61.8%)	3 (21.4%)	
M	13 (38.2%)	11 (78.6%)	
**ECOG PS**			>0.99
0	33 (97.1%)	14 (100.0%)	
1	1 (2.9%)	0 (0.0%)	
**Primary lesion**			
Absent	34 (100.0%)	14 (100.0%)	
**RAS mutation**			0.51
Absent	13 (39.4%)	7 (53.8%)	
Present	20 (60.6%)	6 (46.2%)	
Unknown	1	1	
**BRAF V600E mutation**			
Absent	32 (100.0%)	13 (100.0%)	
Unknown	2	1	
**MSI-High**			
Absent	29 (100.0%)	12 (100.0%)	
Unknown	5	2	
**Lung metastasis volume**	0.7 (0.4, 1.2)	0.5 (0.2, 2.0)	0.94

ECOG PS: Eastern Cooperative Oncology Group performance status

[1]Median (interquartile range)

[2]Wilcoxon rank sum test or Fisher's exact test
